# Virtual Needle Insertion with Enhanced Haptic Feedback for Guidance and Needle–Tissue Interaction Forces

**DOI:** 10.3390/s24175560

**Published:** 2024-08-28

**Authors:** Mostafa Selim, Douwe Dresscher, Momen Abayazid

**Affiliations:** Robotics and Mechatronics Research Group, Faculty of Electrical Engineering, Mathematics and Computer Science, University of Twente, 7500 AE Enschede, The Netherlands; d.dresscher@utwente.nl (D.D.); m.abayazid@utwente.nl (M.A.)

**Keywords:** liver cancer, CT liver biopsy, haptic feedback, needle insertion simulation, needle guidance

## Abstract

Interventional radiologists mainly rely on visual feedback via imaging modalities to steer a needle toward a tumor during biopsy and ablation procedures. In the case of CT-guided procedures, there is a risk of exposure to hazardous X-ray-based ionizing radiation. Therefore, CT scans are usually not used continuously, which increases the chances of a misplacement of the needle and the need for reinsertion, leading to more tissue trauma. Interventionalists also encounter haptic feedback via needle–tissue interaction forces while steering a needle. These forces are useful but insufficient to clearly perceive and identify deep-tissue structures such as tumors. The objective of this paper was to investigate the effect of enhanced force feedback for sensing interaction forces and guiding the needle when applied individually and simultaneously during a virtual CT-guided needle insertion task. We also compared the enhanced haptic feedback to enhanced visual feedback. We hypothesized that enhancing the haptic feedback limits the time needed to reach the target accurately and reduces the number of CT scans, as the interventionalist depends more on real-time enhanced haptic feedback. To test the hypothesis, a simulation environment was developed to virtually steer a needle in five degrees of freedom (DoF) to reach a tumor target embedded in a liver model. Twelve participants performed in the experiment with different feedback conditions where we measured their performance in terms of the following: targeting accuracy, trajectory tracking, number of CT scans required, and the time needed to finish the task. The results suggest that the combination of enhanced haptic feedback for guidance and sensing needle–tissue interaction forces significantly reduce the number of scans and the duration required to finish the task by 32.1% and 46.9%, respectively, when compared to nonenhanced haptic feedback. The other feedback modalities significantly reduced the duration to finish the task by around 30% compared to nonenhanced haptic feedback.

## 1. Introduction

Percutaneous biopsy is the gold standard procedure for diagnosing liver tumors, and it is performed by inserting a needle into the patient [1]. Preoperative CT scans are preferred over ultrasound for detecting obscure lesions [2]. Scans show the internal structure of the liver and, thus, guide the interventionalist in planning a path toward the tumor. The needle is aligned to the planned path and then inserted axially toward the tumor. Intraoperative CT scans are acquired during the procedure to ensure the needle follows the planned path. The interventionalist reorients the needle to realign it if it deviates from the planned path. Although realignment inside the liver tissue can be sufficient, a retraction and reinsertion of the needle may be required if the deviation from the planned path is too large. This would cause more tissue trauma. Additionally, more CT scans would be needed, leading to increased exposure to hazardous ionizing radiation [2]. Another type of feedback that the interventionalist encounters is haptic feedback.

During CT-guided needle insertions, interventionalists experience kinesthetic feedback that is caused by needle–tissue interactions via forces and torques. Kinesthetic feedback increases interventionalists’ awareness of the amount of force that is being exerted on the tissue, helping to mitigate excessive force on tissue and aiding in recognizing tissues with different stiffnesses [3]. However, it is hard to recognize deep structures by solely relying on needle–tissue interaction forces [4]. Therefore, multiple CT scans are necessary to ensure that the needle reaches the tumor target deep inside the tissue. Consequently, the interventionalist and the patient are exposed to more hazardous radiation. Teleoperation enables interventionalists to perform surgical procedures away from hazardous radiation [5]. This prompts an investigation into the various types of haptic feedback that could assist the interventionalist during teleoperation, enabling them to perform the insertion efficiently with less reliance on non-real-time visual feedback.

### 1.1. Related Work and Contributions

The interventionalist can control a surgical robot through a source device while guiding a needle with force feedback assistance [6] or through the combination of kinesthetic and vibrotactile feedback [4]. Our study is focused on kinesthetic feedback providing forces through a single haptic interface.

One of the types of kinesthetic feedback is proximity-based haptic feedback, which has been used to increase resisting forces to slow down the operator as the tool moves closer to the target and eventually stops the tool before penetrating critical regions. It can reduce interaction forces, leading to safer interventions [7,8]. Others have focused on relaying needle–tissue interaction forces to the user. The needle experiences three main types of forces as it penetrates through soft tissue: stiffness and cutting forces at the tip and friction on the shaft [9]. Needle-tip force feedback is necessary to detect deep tissue interfaces [10] as tip forces change the most with puncture events and changes in tissue structures [4,11]. However, tip forces are often overshadowed by relatively significant friction forces [12]. Perceiving different deep tissue structures during needle insertion is made possible by scaling the tip forces and reducing the overshadowing effect of friction forces [12]. This idea is implemented by integrating sensors that separate tip and shaft forces such as coaxial sensor [12,13] or needle-tip force sensors [11,14,15]. Furthermore, magnified haptic feedback shows better results in discriminating soft tissues compared to direct hand interactions [16].

Aggravi et al. [4] compared multiple haptic feedback modalities by providing guidance and tissue cutting-force information via kinesthetic and cutaneous interfaces both individually and simultaneously during ultrasound needle insertion experiments. They concluded that users performed with the highest accuracy when implementing force feedback for guidance and cutaneous vibrotactile feedback for perceiving cutting forces [4]. However, they did not investigate a haptic modality that provides both guidance and tool–tissue information simultaneously on a single haptic interface (kinesthetic or vibrotactile). Abayazid et al. also showed that the targeting accuracy improved by nine times compared to manual insertions when guiding visual fixtures and vibratory haptic feedback were combined to provide guidance to steer a needle under ultrasound [17].

On the other hand, other studies have shown that the effect of haptic feedback can be insignificant or cause undesirable side effects during needle insertion tasks. Gerovich et al. reported that force feedback due to tissue stiffness and damping is only beneficial when visual feedback of tissue deformations is absent [18]. Wang et al. showed that, even though users could perform better with haptic feedback, it could cause more cognitive overload and muscle fatigue [19].

In this study, we investigated the effect of enhancing visual and haptic feedback on users’ performance in targeting a tumor in 5 DoF virtual CT-guided needle insertions. The users experienced 3 DoF forces via the haptic device for guiding the needle and sensing axial needle–tissue interaction forces. The enhanced force feedback lies in increasing the intensity of stiffness forces guiding the needle and magnifying the axial tip forces during tumor interaction. Visual feedback was achieved via an interior scan of a virtual fixture simulating a liver CT scan that showed the needle pose relative to the target. This scan could be provided either upon user request or enhanced through real-time feedback. We aimed to give insights into the effect of these feedback configurations by performing a statistical analysis of the results. The main motivation for this work was to enable efficient teleoperated needle targeting and to decrease reliance on CT scans. In addition, we aimed to use this work as a potential training platform to reduce the performance gap between novices and experts by utilizing haptic feedback, since haptic feedback is proven to support clinicians in training [20]. It is still not clear from other works how various combinations of force feedback configurations for guidance and sensing needle–tissue interaction, rendered on a single haptic interface, affect user performance in targeting the tumor and their degree of reliance on the CT scan.

The main contribution of this study is providing insights into the following:

1. The effect of enhancing force feedback in the axial and/or radial directions of the needle on the users’ ability to efficiently perceive needle–tissue interaction and guide the needle to the target, respectively.
2. The effect of enhanced force feedback on the degree of reliance on CT scans (visual feedback) to guide the needle to the target.
3. The effect of real-time visual feedback compared to enhanced real-time haptic feedback on user performance.

### 1.2. Scope of the Study

There are different aspects that were explicitly not part of this study. Torque feedback on the needle from tissue interaction and the possibility of controlling the orientation of the needle within the liver tissue was not implemented into the controller. We restricted the system to translation force feedback due to device limitations. In addition, we did not include needle deflection or tissue deformation models. This means that the needle was modeled as a stiff shaft and that the various tissue structures did not displace relative to each other due to deformation.

## 2. Materials and Methods

### 2.1. Setup and Simulation Environment

**Figure 1:**
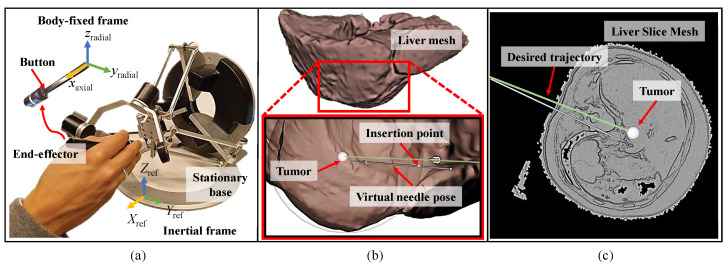
(**a**) The Omega.6 haptic device for controlling the pose of the virtual needle via its end-effector, (**b**) a liver mesh exterior view with a gray arrow representing the pose of the needle, and (**c**) a liver slice showing the interior structure of the liver highlighting the tumor target.

We used the Omega.6 haptic interface (Force Dimension, Nyon, Switzerland), as shown in Figure 1a, which has 6 DoF of motion and provides haptic feedback on the 3 DoF of linear motion. It has a pen-shaped end-effector suitable for controlling a virtual needle. The device was connected to a laptop with an open-source Linux-based Ubuntu 20.04 with kernel number 5.15. The needle insertion simulator was implemented on the Robot Operating System (ROS Noetic) and visualized using ROS Visualization (RViz).

The simulation environment in Figure 1b shows a 3D mesh of a human liver constructed from a CT dataset (lits17) [21] and a 3D arrow to visualize the pose of the needle. Spherical RViz markers were designed to simulate the boundaries of the mechanical properties of the liver capsule, liver tissue, and tumor. The tumor target is a 2.5 mm diameter sphere, simulating a hepatocellular carcinoma (HCC) tumor of a small size range [22]. The mechanical properties overlap with the entire insertion region on the liver mesh to transfer the needle–tissue interaction forces to the user while inserting the needle into the various tissue structures through the haptic device. A slice of a liver mesh constructed from a CT scan [23] was used to mimic a CT scan, illustrating the internal liver structures, as shown in Figure 1c.

### 2.2. Visual Feedback Strategy

The visual feedback (liver slices) showed the internal movement of the virtual needle, projected on the liver slice plane relative to the tumor. The updated pose is either shown upon the user’s request, as normally performed in CT-guided interventions, and then recorded as one scan, or they can be enhanced and shown continuously in real time without needing the user’s request. In Section 2.4, we explain how they were used for each experimental scenario.

### 2.3. Haptic Feedback Strategy

In this study, the user experienced forces in 3 DoF through a hybrid controller running at 1 KHz combining position and force controllers in the radial and axial directions of the virtual needle, respectively, as shown in Figure 1. Radial forces provided guidance, while axial forces were used to sense needle–tissue interaction forces, enabling the distinction between different tissue layers. However, during needle insertion procedures, interventionalists adjust the needle orientation around a point near the incision to maintain proper alignment with the tumor while receiving torque feedback. Since the required circular motion inside the tissue to align the needle is minimal, the motion performed by the user can be linearized and approximated to a translation motion. Consequently, radial force feedback was provided at the point where the user holds the needle. These radial movements were carried out in the end-effector body-fixed frame, as shown in Figure 1. This approach provided the necessary guidance cues for the user to achieve the desired needle alignment. Once the needle penetrates the virtual tissue fixture, the needle angle is maintained for more controlled insertion.

#### 2.3.1. Radial Force Feedback

Radial forces acting on the haptic device handle ensured that the needle predominantly moved axially when it was inserted into the tissue. A proportional–derivative (PD) position controller was implemented to constrain radial movement and to mitigate user tremors while inserting. Equation (1) represents the spring-damper model of radial forces,

(1) $$f_{\mathrm{radial}}=k_{p}d-k_{d}v,$$

where $f_{\mathrm{radial}}\in R^{2}$ represents radial forces on the virtual needle in the z–y plane of the body-fixed frame of the needle, as shown in Figure 1. $k_{p}$ is a constant proportional gain and $d\in R^{2}$ is the orthogonal vector from the incision point to the virtual needle with some tolerance *t* to move freely in a small cylindrical capsule. *t* enabled the user to realign the needle with the target. The incision point is the location where the needle penetrates the tissue. $k_{d}$ is a constant derivative gain and $v\in R^{2}$ is the radial velocity vector of the needle.

The magnitudes of the stiffness parameters of the radial forces dictated whether the feedback was compliant or enhanced. Compliant force feedback gave more freedom to the user to correct for misalignment, and enhanced force feedback enabled the user to insert the needle axially with reduced tremor by increasing the constraints in the radial direction. The magnitudes for the stiffness parameters are shown in Section 2.4.

#### 2.3.2. Axial Force Feedback

**Figure 2:**
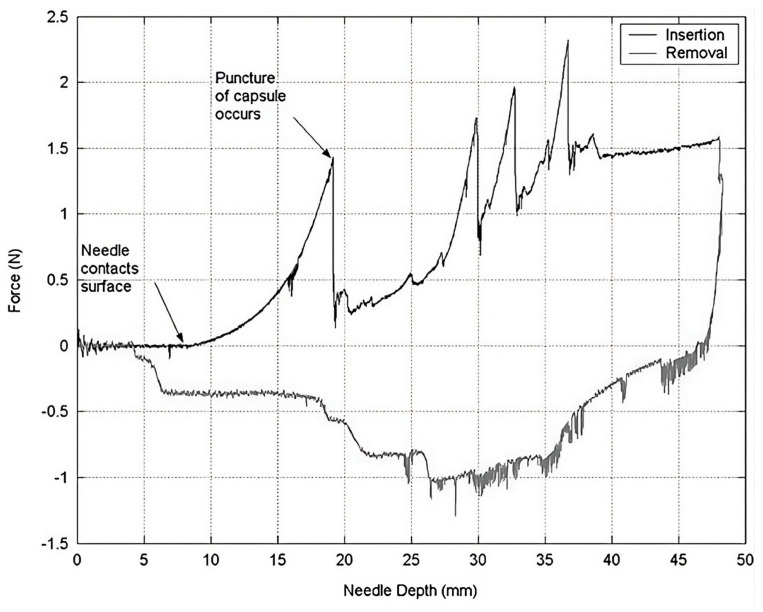
Axial force measurement of the needle while being inserted into a bovine liver (© 2004, IEEE) [9]. The force profile generated shows the nonlinear stiffness when the needle started to contact the surface, followed by a drop in forces after puncture. Subsequently, the force increased due to frictional forces, with some peak forces caused by contact with internal structures during insertion, such as blood vessels. The force profile from the simulation in this study, depicted in Figure 3, should have a similar shape.

The implemented axial force model of the liver tissues adopted the force model proposed by Okamura et al. [9]. They proposed three different models, namely, a nonlinear spring model for stiffness forces $f_{\mathrm{stiffness}}$, the Karnopp model for friction force $f_{\mathrm{friction}}$, and a constant for cutting forces $f_{\mathrm{cutting}}$ for a given velocity. The total axial force $f_{\mathrm{axial}}$ acting on the needle in the x-axis of the body-fixed frame is shown in Equation (2). Their characterization was based on a needle insertion into a bovine liver while measuring axial forces on the needle. The force profile is shown in Figure 2. However, the force spikes that occurred after puncture, due to contact with vessel structures, were not included in the model.

(2) $$f_{\mathrm{axial}}=f_{\mathrm{stiffness}}+f_{\mathrm{cutting}}+f_{\mathrm{friction}}.$$

**Figure 3:**
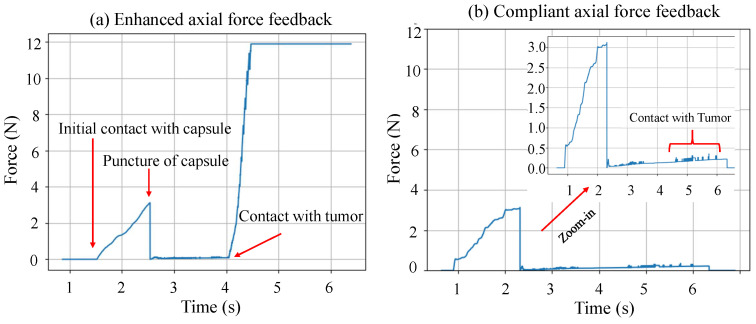
Axial force measurement of the needle as it penetrates different liver structures with (**a**) enhanced and (**b**) compliant force feedback.

In this study, axial force feedback provided to the user was divided into two parts; needle tip and shaft forces.

(a) Needle-tip forces:

These are the stiffness and cutting forces. To simulate the stiffness-related forces of the liver capsule on the tip, a nonlinear spring model adopted from [9] was implemented in Equation (3),

(3) $$f_{\mathrm{stiffness}}=k_{\mathrm{stiff}}{\left(x_{\mathrm{init}}-x_{\mathrm{cur}}\right)}^{2}+k_{\mathrm{stiff}}\left(x_{\mathrm{init}}-x_{\mathrm{cur}}\right),$$

where $k_{\mathrm{stiff}}$ is the stiffness of the liver tissue, $x_{\mathrm{init}}$ is the initial point of contact of the virtual needle tip with the liver tissue, and $x_{\mathrm{cur}}$ is the current depth of the tip.

Cutting forces were modeled as a constant for a given tissue cut by a constant velocity needle, as suggested in [9]. It is inconclusive whether the velocity has an effect on the cutting forces [24]. In this study, cutting forces were assumed to be a viscous-friction model to account for different velocities of insertion. These forces act on the tip to penetrate through the tissues and are governed by Equation (4),

(4) $$f_{\mathrm{cutting}}=vk_{\mathrm{cut}},$$

where $k_{\mathrm{cut}}$ is the coefficient of cutting forces and *v* is the axial velocity of the needle.

(b) Needle-shaft forces: These are the friction forces $f_{\mathrm{friction}}$ adopted from [9] and implemented in Equation (5),

(5) $$f_{\mathrm{friction}}=\left\{\begin{array}{cc} f_{\mathrm{static}}, & \mathrm{if}\;v<\epsilon \\ f_{\mathrm{dynamic}}+f_{\mathrm{damping}}, & \mathrm{otherwise} \end{array}\right.$$

The friction forces were divided into two stages. The first stage involved constant stiction forces, $f_{\mathrm{static}}$, when the needle moved at an insignificant velocity less than a predefined threshold, ϵ. The second stage involved a position-dependent damping and dynamic friction when the needle moved at a velocity *v* above the set threshold. The dynamic friction $f_{\mathrm{dynamic}}$ is a constant with a magnitude slightly less than that of the stiction forces, while the damping forces are represented in Equation (6),

(6) $$f_{\mathrm{damping}}=cv\left(x_{\mathrm{penet}}-x_{\mathrm{cur}}\right),$$

where $f_{\mathrm{damping}}$ is the damping force, *c* is the damping coefficient, and $x_{\mathrm{penet}}$ is the point of penetration of the tip into the liver tissue.

The axial and radial force vectors, represented in the body-fixed frame (B) on the Omega.6 end-effector, were transformed to the inertial (reference) frame (I) of the haptic device using the rotation matrix $R_{B}^{I}\in \mathrm{SO}\left(3\right)$. The total force $f_{\mathrm{total}}$$\in R^{3}$ command was given to the Omega.6 in the inertial frame as shown in Equation (7):

(7) $$f_{\mathrm{total}}=R_{B}^{I}\left[\begin{array}{c} f_{\mathrm{axial}}\\ f_{\mathrm{radial}} \end{array}\right].$$

**Figure 4:**
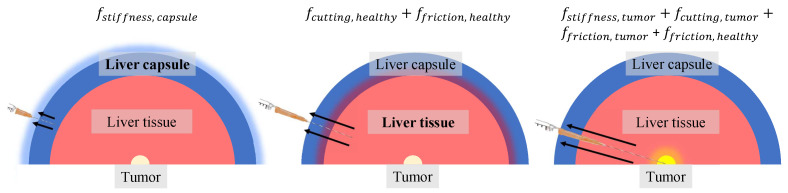
A sketch of the different layers of liver tissues, starting with the liver capsule, then, finally, the tumor at the core. This sketch resembles a cross-section of the three spherical markers carrying the mechanical properties of each phase of insertion. The corresponding axial forces are shown for each insertion phase as the needle encountered different types of tissue.

The parameters of the liver model were tuned to generate a force profile similar to the one shown in Figure 2. As previously mentioned, the liver model consisted of three layers: the liver capsule, liver tissue, and tumor. Each layer had distinct mechanical properties, resulting in different needle interactions as it transitioned from one layer to the next. Figure 4 shows the arrangement of these layers to simulate the various phases of insertion and their corresponding axial forces.

Figure 3 shows that forces increased as the needle penetrated the capsule, then dropped significantly after puncture. As the needle cut through the liver tissue, the forces gradually increased to a magnitude of around 0.3 N due to depth-dependent damping and cutting forces. A similar model to the liver tissue is implemented for the tumor target, but with higher magnitudes for the biomechanical parameters. The magnitudes of these parameters determined whether the feedback is compliant or enhanced. For enhanced feedback, the tumor’s stiffness was significantly amplified compared to compliant feedback to recognize the change in tissue structure. The cutting and friction forces were slightly amplified for enhanced feedback compared to compliant feedback. Therefore, the user experienced a significant increase in axial forces during needle interaction with the tumor when enhanced haptic feedback was enabled compared to compliant haptic feedback, as shown in Figure 3. The magnitudes of the biomechanical parameters selected for each tissue structure are presented for the different cases of force feedback in Section 2.4.

#### 2.3.3. Modes of Operation

In order to maintain the position of the needle when the user was not inserting it, a button on the end-effector of the haptic device was used to toggle between two different states. The first state involved the user inserting the needle while pressing the button, whereas the second state occurred when the needle no longer needed to be moved, such as when the target was reached and the button was released. Equation (8) characterizes the PD controller implemented for the second state to keep the handle position stable.

(8) $$f_{maintain}=k_{p}e-k_{d}v,$$

where $f_{maintain}$$\in R^{3}$ is the force expressed in the inertial frame that kept the end-effector in position and rejected disturbances. The proportional and derivative gains, $k_{p}$ and $k_{d}$, are the same magnitude as in Equation (1), and $e\in R^{3}$ is the deviation vector of the needle’s tip from the latest position before releasing the button. $v\in R^{3}$ is the linear velocity of the needle.

### 2.4. Experimental Design

**Figure 5:**
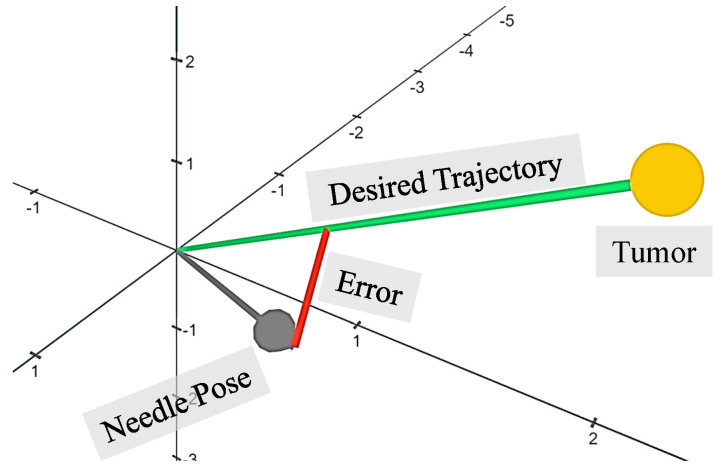
The trajectory tracking error is the shortest distance between the tip of the needle and the desired trajectory. It is orthogonal to the desired trajectory which starts from the desired incision point to the tumor target. The graphical representation does not reflect the true dimensions of the needle and the tumor.

The experiments assessed participants’ performance using the proposed haptic and visual feedback algorithms. The performance criteria evaluated in these experiments were the following:

(a) The number of scans needed throughout the experiments.

(b) The duration to complete the task with a maximum limit of 90 s per trial. The time limit was set to restrict the number of reinsertions performed by participants to achieve maximum accuracy. Therefore, the experiment was controlled with a predefined maximum duration per trial, which was determined empirically through preliminary tests.

(c) Trajectory tracking of the needle tip, a measure of its deviation from the desired trajectory, which starts from the desired incision point to the tumor’s centroid. It is the shortest distance between the needle tip and the desired trajectory, as shown in Figure 5. The average error across the entire trajectory was recorded.

(d) Targeting accuracy, final Euclidean displacement error of the needle tip to the target centroid.

Twelve participants (9 males and 3 females, ages from 24 to 32) without clinical experience took part in the study. All of them were right-handed and had no or little experience with haptic feedback systems. They did not report any visual or haptic perception deficits. They were introduced to the setup and had approximately 15 min before the experiments to become acquainted with it and understand the task they needed to perform. They were asked to finish the task as quickly and efficiently as possible while notifying them about the time. Every participant performed the experiment for five scenarios of haptic and visual feedback modalities without prior knowledge of the haptic feedback that they were going to experience. The five experimental scenarios are described in detail as follows:

- Baseline (BL) scenario, compliant radial force feedback with intermittent visual CT-like scan feedback provided upon request. The axial forces were not enhanced and are similar to what the interventionalist would feel in an insertion procedure. The simulated tumor exhibited a slightly greater magnitude in terms of friction and cutting forces compared to liver tissue.
- Real-time (RT) scenario, real-time and continuous visual feedback. The participant did not need to request scans, since they were automatically displayed. Axial and radial forces were implemented exactly like the baseline scenario.
- Enhanced sensing (ES) scenario, enhanced axial forces. The axial forces were magnified when the needle made contact with the tumor. All other aspects of haptic and visual feedback were provided in the same way as the baseline scenario.
- Enhanced guidance (EG) scenario, enhanced radial forces. The radial forces were amplified throughout the entire scenario. All other aspects of haptic and visual feedback were provided in the same way as the baseline scenario.
- Enhanced sensing and guidance (ESG) scenario, enhanced axial and radial forces. The axial and radial forces were amplified (combination of EG and ES). Visual feedback was provided upon request.

All participants started with the baseline (BL) experimental scenario. After the baseline (BL) scenario, the order of scenarios for the first six participants was RT, ES, EG, and then ESG. The remaining six participants experienced the order ESG, EG, ES, and then RT to avoid training bias towards either enhanced visual feedback or enhanced haptic feedback. Each scenario was performed three times to attain repeatability. The average of each performance metric was calculated.

Table 1 presents the values assigned to the tissue properties in each experimental scenario. The magnitudes of each parameter were selected to achieve a realistic needle insertion into the liver, with a force profile similar to that shown in Figure 2. Additionally, the parameters for axial forces experienced by users when they reach the tumor were magnified for ES and ESG, while the radial parameters used for guidance were increased for EG and ESG.

**Figure A1:**
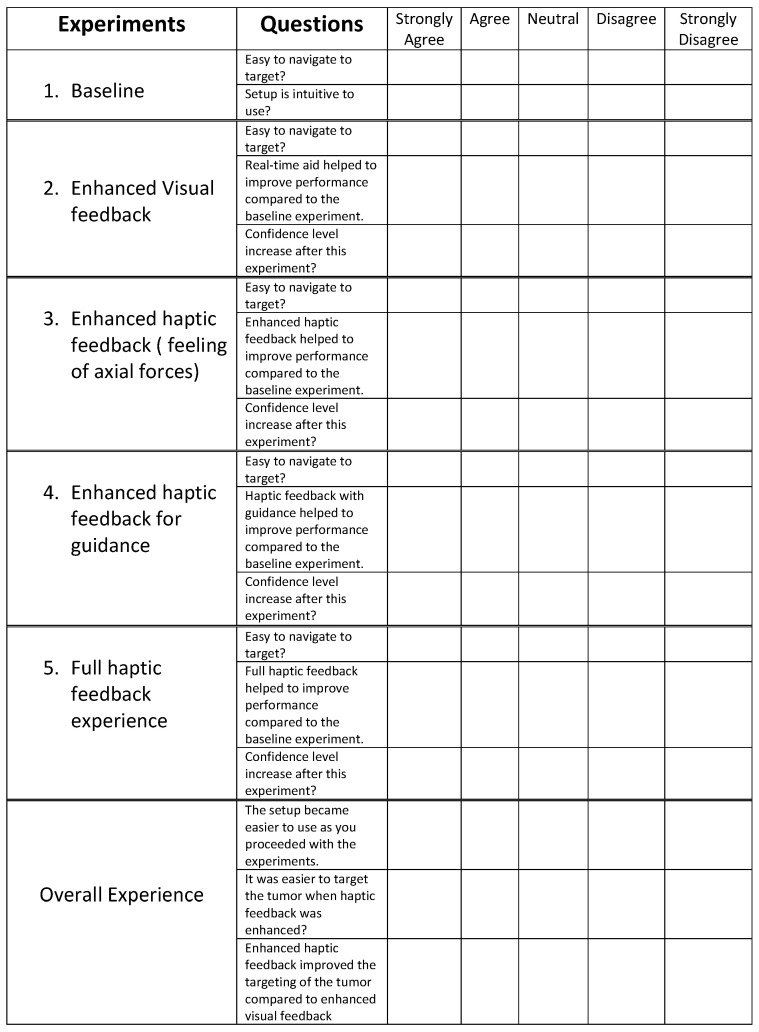
The questionnaire provided to the participants. It is divided into questions asked after each experimental scenario and questions asked after finishing the whole experiment.

Participants started each trial without prior knowledge of the type of feedback they would encounter. During the trials, participants requested scans of liver slices whenever they needed an updated image of the latest pose of the needle relative to the tumor target. They also selected their preferred view to facilitate task performance. They were given a maximum of 90 s per trial. After conducting three trials of each scenario, they answered questions, as shown in Figure A1 in the Appendix A section, about the intuitiveness of the experiment and the difficulty of finishing the task. At the end of the study, participants completed a questionnaire (Figure A1 in Appendix A) evaluating the value of enhanced haptic feedback compared to enhanced visual feedback and commented on the learning curve associated with performing the task.

## 3. Results

### 3.1. Performance Evaluation

**Figure 6:**
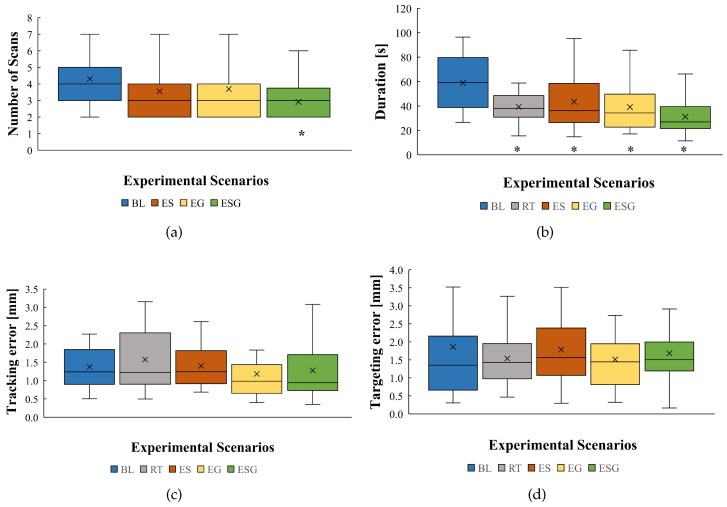
The box plots show the mean, median, minimum, and maximum of all participants’ trials for each experimental scenario. The five scenarios are baseline (BL), real-time (RT), enhanced sensing (ES), enhanced guidance (EG), and, finally, enhanced sensing and guidance (ESG). Each plot shows the following measured metrics: (**a**) number of scans, (**b**) time needed to finish the task, (**c**) average trajectory tracking error, and (**d**) tumor targeting error. The “*” sign is added to the scenarios where the user achieved a significant result in a specific metric.

In this section, we present the data collected from all participants’ experiments that show the number of scans required, duration to finish the task, trajectory tracking, and targeting accuracy. Figure 6 shows the results of the average performance of all participants’ trials for each experimental scenario. The various scenarios are compared to the baseline scenario, showing a * sign if it is a significant result. The outliers are not shown on the plot but they are considered in the calculations.

Number of scans: Figure 6a shows that the ES scenario reduced average scans by 17.2%. Similarly, the EG scenario reduced average scans by 14.2%. Finally, the ESG scenario reduced average scans the most, by 32.1%.

Duration: Figure 6b shows that the RT and EG scenarios had similar effects on reducing the average duration to finish the task by 33.2% and 33.6%, respectively. The ES scenario reduced average duration by 26%. Finally, the ESG scenario reduced average duration the most, by 46.9%.

Trajectory tracking accuracy: Figure 6c shows that the scenario RT increased average trajectory tracking error by 12.7% and the error for the ES scenario only had a slight increase by 1.41%. However, the average tracking error was reduced in the scenarios EG and ESG by 14.5% and 7.2%, respectively.

Targeting accuracy: Figure 6d shows that the scenarios RT and EG had similar effects on reducing average targeting error by 17.2% and 18.8%, respectively. Scenarios ES and ESG barely reduced the average error, by 4.3% and 9.7%.

A one-way ANOVA test was applied to the results, including Tukey HSD (honestly significant difference) for pairwise comparisons. We used a 0.05 significance level. The result was significant for the number of scans with *p* = 0.034. There was a significant difference only between experimental scenarios BL and ESG with *p* = 0.0179. The result was also significant for the duration with *p* <$10^{-5}$. The largest significant difference was between scenarios BL and ESG with *p*<$10^{-6}$. There were similar significant differences between scenarios BL and RT and scenarios BL and EG, with a significance level of *p* = $2.9\;\times \;10^{-4}$ and *p* = $2.4\;\times \;10^{-4}$, respectively. The smallest significant difference was between scenarios BL and ES, with *p* ≈ 0.0084. Accuracy and trajectory tracking results were not significant.

### 3.2. Questionnaire Outcome

Table 2 presents the average score from all participants for each question following every experimental scenario, providing an evaluation of their subjective opinions on each scenario. The participants were given five choices, from strongly agree (5) to strongly disagree (1) (5-point Likert scale).

The overall score indicates that participants had the most positive experience in the scenarios ESG and RT, followed by the scenario EG. The scenario ES showed the lowest score. Participants agreed that it was easier to reach the target when haptic feedback was enhanced. Finally, all of the participants agreed that enhanced haptic feedback and enhanced visual feedback helped equally in targeting the tumor.

## 4. Discussion

The experimental scenario ESG, which constituted enhanced haptic feedback for sensing needle–tissue interaction forces and guidance, showed that participants needed the fewest scans and the shortest duration to reach the tumor. This was expected, as participants reported increased confidence in proceeding toward the target without needing extra scans. This was also confirmed by the high score of the ESG scenario in the questionnaire. The EG scenario, featuring enhanced haptic feedback for guidance, showed the highest targeting accuracy and the least trajectory tracking error. However, the statistical analysis indicated that these differences are not significant. Implementing a stiffer spring model in the EG scenario would enhance the user’s ability to follow the trajectory more accurately but could also restrict their ability to make independent decisions when needed.

Although the experimental scenario ESG provided improved target recognition through the combination of enhanced sensing and guidance, enhanced haptic feedback for sensing became too forceful as the needle tip reached the tumor, leading to an increase in targeting error and trajectory tracking error. Participants struggled to maintain the position of the end-effector because of the overemphasized feedback in scenario ES when reaching the tumor, which reflected the lower tracking and targeting scores compared to the other scenarios. This phenomenon could be mitigated by reducing the tumor’s stiffness, providing a subtle alert to the user that the target is reached without significantly affecting the needle’s position. Moreover, tactile feedback could be integrated to signal reaching the target, rather than force feedback through bilateral manipulation. Enhanced haptic feedback for guidance had a more significant impact on improving user performance in targeting and trajectory tracking accuracy than haptic feedback for sensing tissue interaction forces. However, when combined in ESG, the user performed faster and with fewer scans than when they were provided separately in ES and EG. The realism of the proposed needle insertion simulator could be improved by integrating torque feedback, enabling the user to reorient the needle with greater control. Moreover, implementing more sophisticated kinesthetic cues to guide the needle accurately would enable users to make decisions independently without needing to overpower the spring model. Therefore, muscle fatigue caused by various force feedback algorithms needs to be examined to asses the user convenience during needle insertion. Furthermore, it is essential to analyze users’ ability to quickly disregard various haptic cues when making decisions in unexpected situations.

In a real CT-guided scenario, the interventionalist relies on the scan as the ground truth to guide the needle to the tumor, using it to visualize and avoid critical structures. Relying more on haptic feedback instead of the scan, especially without virtual fixtures to protect these structures, may pose safety risks. Therefore, an in-depth investigation is required to assess the impact on procedural safety when relying less on imaging and more on haptic feedback. In addition, needle deflection and tissue deformation models should be implemented to assess their impact on the results. If the haptic feedback algorithm adapts to the dynamic trajectory caused by tumor displacement and needle deflection, the results are less likely to be affected. Therefore, incorporating these models is crucial for obtaining more reliable results that more accurately reflect real-life scenarios.

Implementing enhanced force feedback in a real-world scenario would require a force sensor to measure the tip forces or the total axial forces. In the latter case, a friction model should be implemented to extract tip force measurements, which are then filtered and amplified to improve user perception. Radial force and torque measurements should also be filtered and amplified to guide the needle along the desired trajectory. In the simulation developed for this study, forces were implicitly categorized within the model. Furthermore, stability issues may arise in teleoperated bilateral needle insertion with enhanced force feedback. Therefore, the control architecture should incorporate dissipating energy elements, namely, passivity layers, to ensure system robustness against uncertainties and external disturbances.

## 5. Conclusions

This study proposed a simulation testbed for CT-guided percutaneous needle insertion into the liver, incorporating haptic feedback for sensing needle–tissue interaction forces and guiding the needle toward the tumor. The objective was to investigate how force feedback for sensing and guidance affects user performance during CT-guided procedures. The motivation was to minimize the interventional radiologist’s exposure to radiation, reduce patients’ tissue trauma, and avoid damaging nearby critical structures.

The first contribution of this study was examining whether enhanced haptic feedback in the axial and/or radial direction aids users reach the tumor more efficiently. The results showed that enhanced force feedback in the axial and/or radial direction increased efficiency by significantly reducing task completion time. The second contribution investigated how users’ reliance on CT scans was affected by enhanced haptic feedback. Findings suggest that users relied less on CT scans when force feedback is enhanced in both directions, as they requested significantly fewer scans. Finally, we compared the effects of enhanced visual feedback with enhanced haptic feedback on user performance. The results indicated that both types of feedback had a similar impact on overall user performance and satisfaction. Although the proposed haptic feedback algorithm significantly reduced the number of scans and task duration, improvements in trajectory tracking and targeting accuracy were not significant.

For future work, we aim to integrate torque feedback and involve more participants with clinical experience to assess the feasibility of the setup for biopsy training and teleoperation. Additionally, we plan to investigate how enhanced haptic feedback affects the experiences of interventionalists compared to novices. This study could also be extended to a teleoperation setup involving a robotic manipulator with a force sensor for needle insertion into a physical liver phantom guided by CT imaging. Furthermore, alternative imaging techniques, such as MRI, could be explored for examining soft tissue organs like the breast and prostate. Finally, investigating kinesthetic feedback that enables more compliant shared control is essential, as it provides interventionalists with greater flexibility to disregard haptic cues in unexpected situations.

## Figures and Tables

**Table 1:** The tissue biomechanical properties for each experimental scenario are detailed for radial and axial forces. The parameters include needle-tip forces (stiffness and cutting) and shaft forces (static and dynamic friction, and damping).

Type	Mechanical Properties	Experimental Scenario				
		**BL**	**RT**	**ES**	**EG**	**ESG**
Axial: Liver Capsule	$k_{\mathrm{stiff}}$ (N/m)	100				
Axial: Liver Tissue	$k_{\mathrm{cut}}$ (Ns/m)	5				
	$f_{\mathrm{dynamic}}$ (N)	0.8				
	$f_{\mathrm{static}}$ (N)	1				
	*c* (Ns/m)	10				
Axial: Tumor	$k_{\mathrm{stiff}}$ (N/m)	0	0	1000	0	1000
	$k_{\mathrm{cut}}$ (Ns/m)	7	7	10	7	10
	$f_{\mathrm{dynamic}}$ (N)	0.9	0.9	1.4	0.9	1.4
	$f_{\mathrm{static}}$ (N)	1.1	1.1	1.8	1.1	1.8
	*c* (Ns/m)	10.5	10.5	15	10.5	15
Radial	$k_{p}$	100	100	100	1000	1000
	$k_{d}$	30	30	30	70	50

**Table 2:** Questionnaire results: The average scores from all participants ranged from 5 (strongly agree) to 1 (strongly disagree). The overall score is the average of all questions, reflecting the degree of satisfaction with each experimental scenario.

Scenarios	RT	ES	EG	ESG
Improved performance compared to baseline	4.3	3.7	4.3	4.5
Easy to navigate to target	4.5	3.6	4.2	4.4
Confidence level increase after experiment	4.5	4	4.1	4.5
Overall score	4.4	3.8	4.2	4.5

## Data Availability

Data unavailable due to privacy restrictions.

## Abbreviations

The following abbreviations are used in this manuscript:

CT	Computed tomography
ROS	Robot Operating System
HSD	Honestly significant difference
DoF	Degrees of freedom
PD	Proportional–derivative
